# Stress Granules in the Viral Replication Cycle

**DOI:** 10.3390/v3112328

**Published:** 2011-11-18

**Authors:** Hilda Montero, Vicenta Trujillo-Alonso

**Affiliations:** 1 Instituto de Salud Pública, Universidad Veracruzana, Av. Luis Castelazo Ayala s/n, Col. Industrial Ánimas, 91190, Xalapa, Veracruz, México; 2 Instituto de Biotecnología, Universidad Nacional Autónoma de México, Av. Universidad 2001, 62210, Cuernavaca, Morelos, México

**Keywords:** stress granules, stress, PKR, eIF2

## Abstract

As intracellular parasites, viruses require a host cell in order to replicate. However, they face a series of cellular responses against infection. One of these responses is the activation of the double-stranded RNA (dsRNA)-activated protein kinase R (PKR). PKR phosphorylates the α subunit of eukaryotic translation initiation factor 2 (eIF2α), which in turn results in global protein synthesis inhibition and formation of stress granules (SGs). Recent studies have shown that SGs can interfere with the replicative cycle of certain viruses. This review addresses how viruses have evolved different control strategies at the SG level to ensure an efficient replication cycle during the cellular stress response triggered by the viral infection.

## 1. Introduction

Viral genomes do not code for all of the components that viruses require in order to complete their replication cycle. Thus, viruses are dependent on diverse factors and conditions in the host cell. However, the process of replication is not easy, because the viral presence within the cell represents a threat that triggers a complex and integrated antiviral response. Interestingly, some viruses have developed strategies that enable them to counteract, tolerate, or even take advantage of this antiviral response, thereby allowing efficient replication.

## 2. A Brief Review of PKR and eIF2

One of the best known mechanisms employed by the cell to restrict viral infection is through double-stranded RNA (dsRNA)-activated protein kinase R (PKR), which is activated by binding to dsRNA, which is generally produced as an intermediary of replication cycle of many viruses [1–3]. PKR is part of the interferon (IFN) response that induces an antiviral state in the infected cell and neighbor cells [2,4]. In the infected cell, PKR phosphorylates the α subunit of eukaryotic translation initiation factor 2 (eIF2α), a modification that blocks the eIF2-GTP-Met-tRNAi^Met^ ternary complex (TC) formation that results in the inhibition of cellular and viral protein synthesis [5]. Thus, by inhibiting the viral protein synthesis, the function of PKR via eIF2α could prevent the formation of new viruses.

Phosphorylation of eIF2α is carried out not only by PKR but also by three other members of the same family of eIF2α kinases that sense specific stress conditions in which the cell is under threat: the general control non-derepressible 2 kinase (GCN2), which responds to the absence of amino acids and other nutrients; the heme-regulated kinase (HRI), which is activated under conditions of intracellular iron deficiency or heat shock; and the PKR-like endoplasmic reticulum kinase (PERK), which is activated by an accumulation of unfolded or misfolded proteins. In all of these cases, activation of these kinases induces the phosphorylation of eIF2α, thereby blocking the cellular translation process [6–8].

Given the role of PKR, many viruses, such as vaccinia, influenza, and poliovirus (PV), employ mechanisms to avoid its activation or to block its function [9–11]. However, the presence of a virus within a cell generates many cellular changes that trigger not only the activation of PKR but also the activation of GCN2 or PERK or both [12,13]. Consequently, the viral strategies could operate at the level of eIF2 and not necessarily operate over each one of its kinases. Accordingly, some viruses (herpes simplex virus type 1 [14]) revert the phosphorylation of eIF2α to maintain its function, whereas other viruses (Sindbis virus [15] and cricket virus [16,17]) employ translational mechanisms independent of eIF2. In addition, eIF2 is a cell death regulator that makes it an important control target for those viruses that inhibit or stimulate cell survival [18,19]. One of the disadvantages of viral control at the level of eIF2, but not control over each one of the eIF2α kinases, could be the induction of several cellular responses like IFN by PKR or Unfolded Protein Response by PERK. Therefore, it is not surprising that the same virus regulates the cellular antiviral response at more than one level with different goals, and this could depend on the needs that arise during the viral cycle and could be related to whether a chronic or acute infection is established.

## 3. Stress Granules

PKR and eIF2 are not the only factors that limit the production of new viral particles. The formation of stress granules (SGs) was recently described as being part of the cellular response to stress generated by viral infection [20]. The SGs are aggregates that contain preinitiation complexes, a feature that suggests that this is where translation is arrested under different stress conditions [21,22]. Interestingly, the SGs have also been shown to be important regulators of cell death [23].

**Figure 1.**
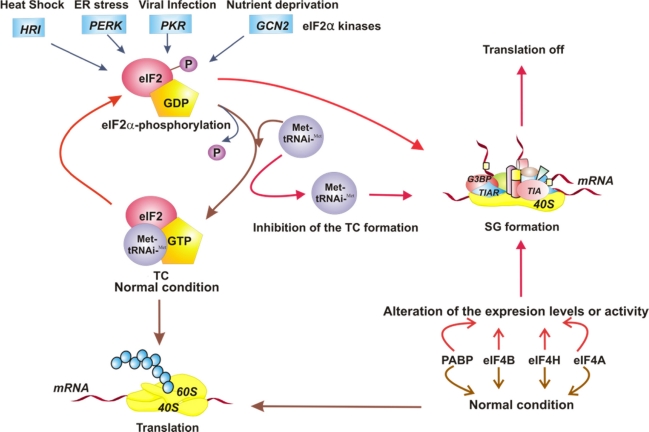
Model of the different stimuli that direct the assembly of stress granules (SGs). The four eukaryotic translation initiation factor 2 (eIF2α) kinases respond to different conditions of intracellular stress, causing phosphorylation of eIF2α and leading to the assembly of SGs. The inhibition of formation of eIF2-GTP-Met-tRNAi^Met^ ternary complex (TC) directs the SG formation also. On the other hand, the alteration of expression level or function of translational factors such as eIF4A, eIF4B, eIF4H, and poly A-binding protein (PABP) induces SG assembly. Under normal conditions, the translation is on. The SG assembly turns translation off.

Initially, it was proposed that SGs are assembled in response to the phosphorylation of eIF2α [24]. However, it has been shown that they are also formed as a consequence of the modification of the expression levels or activity of translational factors, specifically those involved in the initiation phase, such as eIF4A [25], eIF4H, eIF4B, and poly A-binding protein (PABP), or by preventing the formation of the TC by inhibiting the Met-tRNAi^Met^ association [22]. The formation of SGs, therefore, occurs in response to various alterations related to the initiation step of cellular protein synthesis (Figure 1).

To date, the mechanism of SG formation is not entirely understood, and more than 100 genes involved in SG assembly and disassembly have been described [26], suggesting that SG formation is a very complex process. In relation to SG formation, some studies have proposed certain proteins as being responsible for the assembly of these aggregates. Within these effector proteins, which also form part of the SGs, are T-cell intracellular antigen 1 (TIA-1), TIA-1-related protein (TIAR), and Ras-GAP SH3-binding protein (G3BP) [24,27].

It is important to mention that the composition of the SGs varies according to the type of stress [24,28]. Some immunofluorescence microscopy studies suggest that, in addition to being formed by effector proteins, SGs are generally formed by mRNAs; the 40S (but not the 60S) ribosomal subunits; initiation factors such as eIF3, eIF4G, eIF4E, phosphorylated eIF2α [29], and eIF2 [28]; and RNA-binding proteins such as PABP, FMRP (fragile X mental retardation protein), HuR (AU-rich element-binding protein), TTP (tristetraprolin) [21], and caprin-1 [30].

Given that SGs are constituted by preinitiation complexes, it could be expected that the majority of mRNAs are recruited into the SGs. However, mechanisms that determine which mRNAs will be included exist. In two different studies, it was observed that heat shock mRNAs are not found in SGs but were predominantly associated with polysomes [31,32]. Even though the reasons of inclusion or exclusion of mRNAs into SGs have not been established, recent studies show that mRNAs bound to endoplasmic reticulum are not aggregated to the SGs and that the 5′-UTR (5′-untranslated region) plays an important role in their exclusion [33].

The SGs are not aggregated permanently. They disassemble when the cell recovers from a sublethal stress and consequently protein synthesis is restored [21,24,29,34]. The mechanism of disassembly of SGs is also poorly understood, but proteins such as Staufen-1, which binds to dsRNA [35] and microtubules [36], have been described as being important to the disassembly of these aggregates.

## 4. Stress Granules and Viruses

Given their roles as part of the inhibition of cellular protein synthesis and as regulators of cell death, the SGs turn out to be another viral control point downstream of PKR and eIF2. Because each virus undergoes a particular replicative cycle, the impact of SG formation is different for each virus; thus, the viruses could modulate the assembly, composition, or disassembly of SGs according to the replicative cycle. Recent studies have provided valuable information about the relationships between SGs and viral infection. In general, two possibilities exist: the replication cycle of the virus is completed despite the presence of SGs, or the formation of SGs is blocked by viral mechanisms.

Translational regulation during infection with PV is one of the most studied mechanisms, and this virus has been shown to cause a rapid inhibition of cellular protein synthesis through the cleavage of factors eIF4GI, eIF4GII, and PABP [37,38]. In PV-infected cells, the formation of SGs occurs early in the infection and is independent of eIF2α phosphorylation, which happens in a late phase of the infection. The SGs in PV-infected cells are not conventional, because they exclude G3BP, PABP, and eIF4G, and the SGs are assembled next to cell structures containing viral RNA [39,40]. The role of SGs during PV infection is not yet clear; however, there are data showing that SG composition could be important because the incorporation of G3BP into the SGs has a negative impact on PV replication [40]. Another interesting finding is that, in PV-infected cells, the cellular transcription is a modulator of SG assembly since treatment with actinomycin D, an inhibitor of cellular transcription, prevents SG formation [39]. This suggests that the cellular transcription is an intracellular event that may be important to SG assembly in the context of infection. In summary, PV is a virus that modulates the composition of SGs, possibly by interfering with its replicative cycle.

In cells infected by the mammalian orthoreovirus (MRV), SG formation is an early event observed in response to virus entry and does not correlate temporally with eIF2α phosphorylation, suggesting that SGs are formed by a mechanism that is triggered from the first contacts between the virus and its host cell. Interestingly, in the early phase of infection, the SGs include viral core particles but the significance of this remains unclear. SG disassembly is observed as the replication cycle progresses. SG disassembly correlates with an increase of viral protein synthesis [41], indicating that any viral protein may be involved in this process. MRV is, thus, an example of a virus that could regulate the assembly-disassembly of SGs as infection progresses.

The mouse hepatitis coronavirus (MHV) has a replication strategy that makes it tolerant to the presence of SGs as these are assembled during infection in response to eIF2α phosphorylation. Despite this, studies in mouse embryonic fibroblasts (MEFs) that express a mutant unphosphorylatable eIF2α show that the formation of new viral particles is increased, suggesting that the shutoff of protein synthesis and the formation of SGs limit their replication cycle to some extent [42]. Similarly, during infection with respiratory syncytial virus (RSV), eIF2α phosphorylation is observed [43], and the virus also replicates in the presence of SGs. In contrast to MHV infection, during RSV infection, SG assembly has a beneficial effect because, when SG formation is prevented through knockdown of G3BP, viral replication decreases [44].

A strategy very different from SG regulation is presented in cells infected with the human T-cell leukemia virus type 1 (HTLV-1), which can switch SG formation on or off, at its convenience, through the Tax viral protein. Interestingly, Tax shuttles from the nucleus to the cytoplasm in response to several types of stress. When found in the cytoplasm, Tax binds to histone deacetylase 6 (HDAC6) and impedes the formation of SGs, ensuring the synthesis of proteins that may be important for the HTLV-1 replicative cycle. This finding shows that HDAC6 is critical to SG formation. In contrast, when Tax is found in the nucleus, SGs are formed spontaneously [45], and this possibly confers upon cells a resistance to stress by increasing survival [23] and consequently favors the replicative cycle. This type of strategy may allow the establishment of a chronic infection by stimulating cellular events that induce the immortalization and proliferation of infected cells. The study of the pathogenesis of this virus reveals the important role played by HDAC6 as an effector protein of SGs.

**Figure 2.**
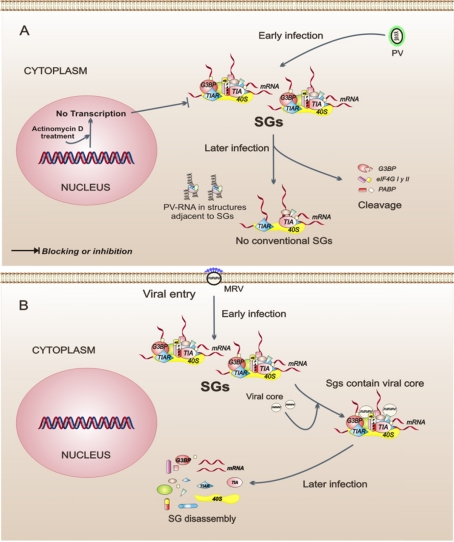

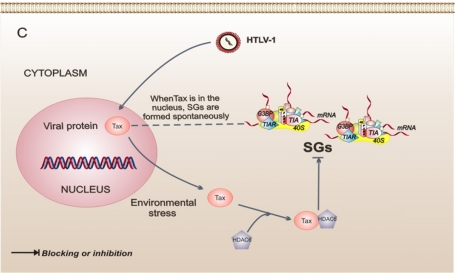
Model of three intracellular events that regulate the assembly of stress granules (SGs) during viral infections. (**A**) Poliovirus (PV) infection stimulates the SG formation early in infection. Later in infection, these SGs have a different composition because they do not contain all of the proteins observed in conventional SGs. Also, in PV-infected cells, cellular transcription is important for the assembly of SGs. (**B**) Orthoreovirus mammalian infection (MRV) induces the formation of SGs in response to virus entry. MRV cores in SGs can be observed at early times of infection. The SGs are dissolved at later times of infection. (**C**) Human T-cell leukemia virus type 1 (HTLV-1) infection. Under intracellular stress, the viral protein Tax shuttles to the cytoplasm from the nucleus, binds to histone deacetylase 6 (HDAC6), and thereby blocks the formation of SGs. The dotted line indicates that, when Tax is in the nucleus of the cell, SGs are formed spontaneously.

Of viruses described here, some can tolerate the antiviral response mediated by SG formation. However, SGs appear to limit the maximum efficiency of the production of viral progeny in the majority of the cases. On the other hand, it should be noted that SG formation could not be necessarily the final event of PKR-mediated phosphorylation eIF2α or of alterations of translational initiation factor. Notably, in the context of viral infection, the SGs can be formed by other stimuli or signaling pathways, such as viral entry (MRV), disturbances of cellular transcription (PV), and the regulation of effector proteins of SGs (HTLV-1) (Figure 2). All of these findings suggest that SG formation could be the results of a great diversity of interconnected intracellular events leading to the same level of regulation.

## 5. Viruses that Interfere with the Assembly of Stress Granules

In the case of cells infected with West Nile virus (WNV) or dengue virus (DV), effector proteins such as TIA-1 and TIAR have a function different from SG formation. It has been shown that both cellular proteins are hijacked by the viral replication complexes and this event can confer to the infected cell resistance to SG formation induced by stressors such as sodium arsenite, a classic inductor of oxidative stress [46]. The presence of TIAR in the replication complexes benefits the viral life cycle of these viruses because, in MEFs that lack TIAR, viral progeny is decreased [47]. Thus, WNV and DV are good examples of viruses that take advantage of cellular antiviral response by using effector proteins of this cellular event.

It was recently shown that PKR phosphorylates eIF2α [48] from the early stages of infection in rotavirus-infected cells. Despite the eIF2α phosphorylation, the formation of SGs is not observed. Rotavirus infection, like WNV and DV infection, confers cellular resistance to the assembly of SGs by treatment with sodium arsenite [49]. Rotavirus has developed a replication mechanism that allows it to overcome eIF2α phosphorylation-mediated translational inhibition and avoids the formation of SGs.

The previous examples of viral infections, in which SG assembly is not observed, show that some viruses have evolved different mechanisms to block the antiviral response at the SG level. Additional experiments are required the elucidation of inhibition mechanisms and the role of SGs in the replicative cycle.

## 6. Conclusions

Despite the knowledge generated in the field of SGs, unresolved issues remain. Because SGs are part of the antiviral response, the viruses regulate this event in order to replicate. It is clear that each virus is related differently to SG formation since the replicative cycle of each virus has different needs. Further study of the molecular mechanisms of SG formation and disassembly, as well as their role and possible regulation, will not only yield information regarding these aggregates but also enable the design of drugs and strategies to control virus replication.
